# Potential health risk of heavy metals in the leather manufacturing industries in Sialkot, Pakistan

**DOI:** 10.1038/s41598-017-09075-7

**Published:** 2017-08-18

**Authors:** Muhammad Junaid, Muhammad Zaffar Hashmi, Yu-Mei Tang, Riffat Naseem Malik, De-Sheng Pei

**Affiliations:** 1 Chongqing Institute of Green and Intelligent Technology, Chinese Academy of Sciences, Chongqing, 400714 China; 2 Environmental Biology and Ecotoxicology Laboratory, Department of Environmental Sciences, Faculty of Biological Sciences, Quaid-i-Azam University, Islamabad, 45320 Pakistan; 3 University of Chinese Academy of Sciences, Beijing, 100049 China; 4 Department of Meteorology, COMSATS University, Islamabad, 45320 Pakistan

## Abstract

This is a systematical report on the potential health risk of heavy metals from the leather industries in Pakistan based on multiple biological matrices of the exposed workers and indoor dust samples. The adverse impacts of heavy metals on the oxidative enzyme and their risks to workers’ health were also explored. Our results indicated that the level of Cr in indoor industrial dust was more than twice, compared to the background household dust. Blood, urine and hair samples of exposed workers showed significantly high concentrations of heavy metals, compared to those in the control group. Superoxide dismutase (SOD) level in the blood samples expressed significant positive correlation with Cr and Ni. Total hazard quotients (HQs)/hazard index (HI) were >1, and Cr (VI) exhibited higher cancer risks than that of Cd in the exposed workers. In addition, the PCA-MLR analysis confirmed that the industrial sections; cutting, shivering/crusting, and stitching were the principal contributors of heavy metals in the biological entities of the workers. Taken together, our results highlighted the occupationally exposed groups would likely to experience the potential health risks due to excessive exposure to the heavy metals from the leather industries.

## Introduction

Leather industry has substantial economic importance; however, it faces more and more criticism because of the toxic waste emissions as a result of leather tanning and processing. Three types of wastes are generated by the leather industries: particulate matter dust (<10 µm), leather fibers (30–1200 µm) and effluents. The dust emitted from the processing of the leather contains 0.1–4.5% of Cr(III), which can cause severe health hazards to the exposed workers^1, 2^. Further, the effluents from the leather industries are ranked as the primary environmental pollutants, because they contain more than 40 different chemicals including heavy metals, acids, and dyes^3, 4^. Therefore, International Agency for Research on Cancer (IARC) listed the wastes from leather industries as human carcinogens, and several studies reported the incidences of blood, bladder, colon, lung, nasal, paranasal sinuses, respiratory tract, and rectum cancers in the exposed workers^2^.

Among heavy metals, Cr is abundantly reported in the leather industries due to excessive use of Cr sulphate [Cr(H_2_O)_5_(OH)SO_4_] salts^5, 6^. Mutagenic properties of Cr depend on various factors such as oxidation states, bioavailability, bio-sorption, ligand mobility, and stability in the environment^7^. The United States Environmental Protection Agency (USEPA) and IARC had categorized Cr (VI) as a human carcinogen^8, 9^. Commercially, Cr is utilized in different occupational settings/industries such as leather tanning, metal finishing, pigment/dyes production, wood preservatives, corrosion inhibition, and glassware cleaning solutions^7, 10, 11^. In consequences, plenty of workers are exposed to Cr-borne fumes, dust, mist, and salts in different occupational settings on a daily basis globally as we have described in one of our previous study^12^.

Occupational exposure to heavy metals other than Cr can also cause enormous health impairments, such as asthma, back pains, bronchitis, chronic dermatitis, chromosomal abrasion, hypertension, hemoglobin changes, metabolic syndrome, DNA damage and even cancer^13–16^. Cd, Pb, and Ni have been classified as mutagenic to wildlife and humans^17^. Chronic exposure to toxic metals can increase the generation of reactive oxygen species (ROS) in the body, which lead to the induction of oxidative stress and cause substantial damage to the cellular components such as lipids, proteins, and DNA^18^. In response, cells naturally activate the immune system in the form of different antioxidants such as superoxide dismutase (SOD), glutathione (GSH) and reduced glutathione (rGSH), *etc*. These antioxidants try to balance the oxidative stress and nullify the toxic impacts of heavy metals through synergistic actions^19, 20^.

Exposure to heavy metals in the leather industries has been implicated as the causative agent for many health hazards as documented by many authors in the past. For example, a cross-sectional survey from Kanpur, India have demonstrated the higher prevalence of medical complaints in the form of hand dermatitis, asthma, and low back trouble in the workers^21^. Rastogi *et al*. reported that the chronic exposure in the leather industries caused pulmonary disorders, such as asthma, chronic bronchitis, and pulmonary tuberculosis^5^. Khan *et al*. reported high disease incidences (skin allergies, bronchitis, and conjunctivitis) in 240 exposed workers in leather tanneries from Sialkot, Pakistan^22^. However, studies that probe the health hazards to the workers using multiple bio-matrices and indoor industrial dust are scarce. Recently, the indoor dust has been proved an excellent marker of indoor heavy metal exposure in the industrial and urban settings^23, 24^.

In biomonitoring studies, the urine and blood samples are widely used as conventional bio-matrices^25^. However, recent studies have mentioned the benefits of using the non-invasive bio-matrices (hairs and saliva) and drawbacks of relying on the classical fluids^24, 25^. Urine and blood samples mainly reflect the recent exposure, therefore, it is not a rationale to assess the chronic exposure to heavy metals using these two bio-matrices^25^. Hairs have higher metal bioaccumulation potential and reflect the past exposure of up to one year due to the slower growth rate (10 mm per month) and the ability of the metal cations to bind with the keratin proteins present in the hair matrix^25^. Moreover, hairs are an easily accessible stable matrix, which makes their collection, storage, and transportation easier^26^. Salvia is also readily collectible diagnostic material with easy accessibility^25^. Although researchers have mixed opinion about the use of saliva and hair samples for biomonitoring, we considered them as mandatory biomonitoring tools to reduce the biases among the bio-matrices and evaluate the contamination status *via a* holistic approach, which ultimately helped us to understand the fate and toxicokinetics of heavy metals in workers.

Sialkot is a populous city and highly prone to environmental pollution because of rapid growth in urban and industrial areas during the last decade^27–29^. This city is famous globally for its leather products, sports goods, and surgical instruments^30^. This is a systematical report from Sialkot on the transport and transformation of heavy metals in different biological and environmental matrices in the leather industries. The objectives of this study include: 1) to monitor toxic heavy metals with elicited concern of Cr in bio-matrices of the exposed workers and indoor industrial dust; 2) to investigate the impacts of industrial environmental contamination on the health status of workers based on cancer and non-cancers risk factors; 3) to investigate potential industrial sources of heavy metals and their percentage contribution in the bio-matrices of the exposed subjects based on PCA-MLR; 4) to measure oxidative stress induced by toxic metals; 5) to evaluate the relationship among heavy metals contamination in indoor industrial dust and bio-matrices of the exposed workers.

## Materials and Methods

### Ethical statement

All the experiments in this study fulfilled and confirmed the mandatory requirements as per the Declaration of Helsinki Ethical Principles for Medical Research Involving Human Subjects^31^. Legal and ethical standards related to this study and experimental protocols were approved by the Ethical Committee, Faculty of Biological Sciences, Quaid-i-Azam University, Islamabad, Pakistan. All of the participants were properly informed about the objectives of this study and only volunteers were selected for sampling after getting the informed consent from all the participants and the approval from the local authorities (Approval ID: 100450068).

### Description of the sampling site and sampling of bio-matrices

**Figure 1:**
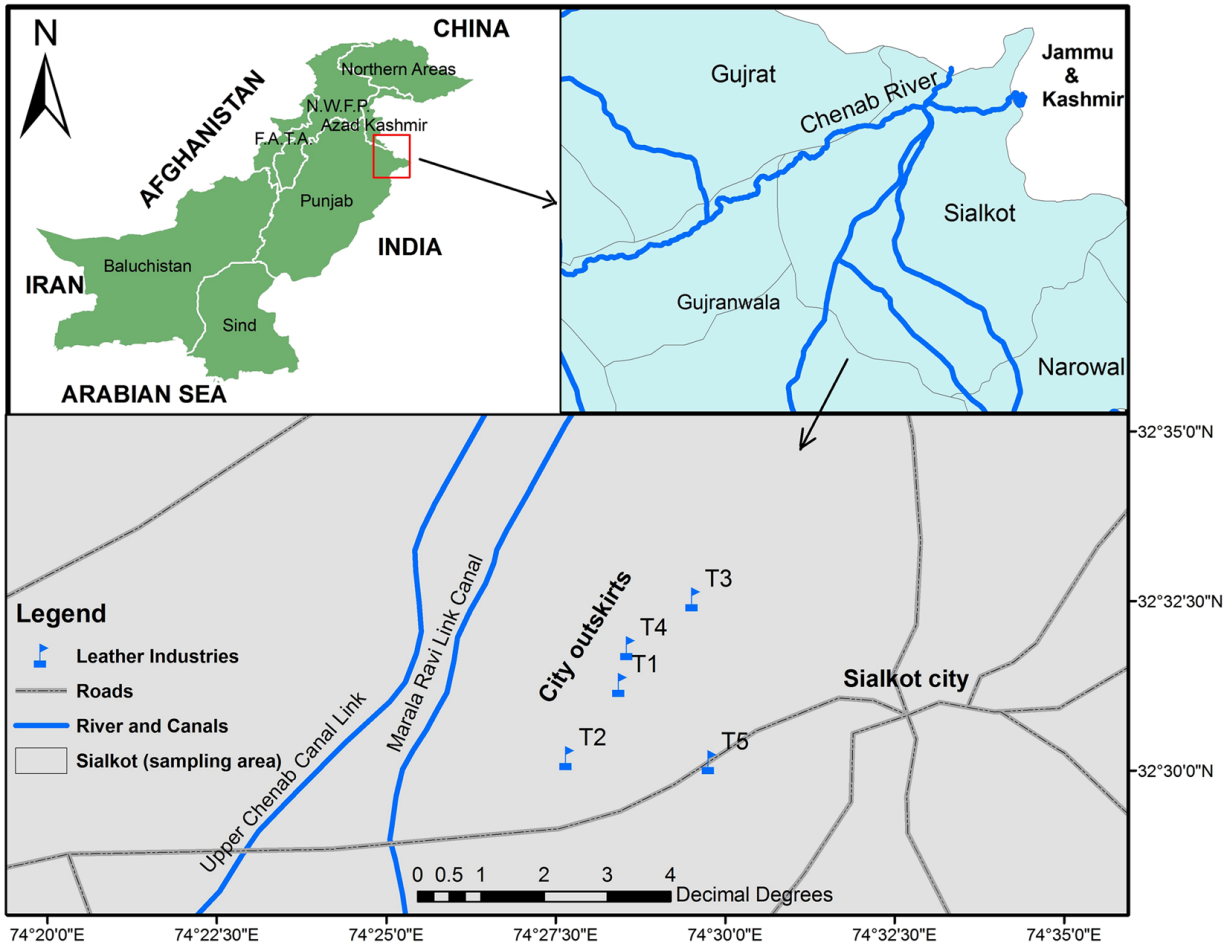
Map of the study area (Sialkot, Pakistan) developed using Arc GIS 9.3 (ESRI, USA) software.

Sialkot city is located between 32°24′N to 32°37′N latitude and 73°59′E to 75°02′E longitude. In Sialkot, the leather industries are concentrated in five main areas; most of them are located in suburban areas. North and Northwestern suburban side of the Sialkot have plenty of registered and unregistered leather industries. Some leather industries are also located within the city. One leather industry was selected from each hub. All the leather industries were given codes such as T1, T2, T3, T4 and T5 **(**Fig. 1 **)**. In close proximity to this sampling area, there are two major canals of the Chenab River, which is a source of freshwater for the region. Therefore, the risk of heavy metal contamination to underground and surface water resources are higher. For biological samples, healthy workers (n = 47, age 20–50 years) were recruited voluntarily for urine, blood, hair and saliva samples. Before sampling, all participants were examined and workers with bleeding in mouth or tooth diseases were not sampled. Psychological and physical stability of the workers is of critical importance while selecting the workers for sampling. The control group (n = 14) was consist of the unexposed population from Cantonment area of Sialkot, with clean and hygienic environmental conditions. Further description of methods for sampling, sample preparation, and digestion of biological matrices and environmental samples are presented in the supporting information (Text S1). The details about the digestion conditions of biological and environmental samples are given in the Table S2.

### Estimation of heavy metals in dust and bio-matrices

Flame atomic absorption spectroscopy (FAAS) (Varian Spectra AA-240) was used to quantify the heavy metals (Cr, Cd, Ni, Cu, Mn, Fe, Pb, and Zn) in the dust and bio-matrices following the methods described somewhere^25^. Calibration of the instrument was performed using a separate set of standards for dust samples (3.125, 6.25, 12.5, 25, and 50 ppm) and bio-matrices (5, 2.5, 1.25, and 0.62 ppm). After calculation of the dilution factor, the heavy metal concentrations were presented with following units: µg/L for urine, blood, and saliva, mg/g for dust, and µg/g for hairs. The instrumental conditions of FAAS for measurement of the heavy metals are presented in the Table S3.

### Quality assurance and quality control (QA and QC)

Strict compliance with quality assurance and quality control methods was implicated throughout sampling and analysis. All of the glasswares and containers were soaked in 10% HNO_3_ and then washed with distilled water and oven dried prior to use. Analytical grade (Merck, Germany) chemicals and reagents were used. For quality assurance (QA) of analytical methods reagent blanks and triplicate samples were analyzed to reduce the biases. The recoveries were calculated in the range of 84–105% for all the investigated metals (Table S3). Quality control (QC) was implicated through analyzing the certified reference material (CRM). Human saliva and urine CRMs were obtained from National Institute of Environmental Studies, Japan. CRM of whole blood was obtained from External Quality Assessment Scheme, Canada and CRM for hair was purchased from National Research Centre, Beijing, China. The details related LOQs, LODs, accuracy, precision, and recoveries of the investigated heavy metals are presented in the Table S3.

### Heavy metals source apportionment in bio-matrices using PCA-MLR

Principal component analysis (PCA) is well-acknowledged source apportionment technique. However, it only provides qualitative information about the potential sources through principal components without interpreting the quantitative information. Principal component analysis-multiple linear regression (PCA-MLR) provides the quantitative information about the sources, which can help to disintegrate the overlapping components/sources. In this study, MLR analysis was applied between PCA scores and pollution index to elucidate the relationship among individual heavy metal sources to the rest of the potential heavy metal sources^32^. This PCA-MLR method was previously described by Thurston and Spengler^33^. The final percentage contributions of industrial sections were calculated using the following Eq. 1 devised by Wu *et al*.^32^.

1 $${\rm{Percentage}}\,{\rm{contribution}}\,{\rm{of}}\,{\rm{a}}\,{\rm{specific}}\,{\rm{section}},i=(\frac{{\rm{B}}i}{\sum Bi})\times 100$$

where B*i* = regression coefficient for the specific factor (contribution of single industrial section) and $\sum Bi$ is the sum of the all the regression coefficients (overall input through all the sections).

### Human health risk calculation

#### Non-cancer health risk

The non-cancer health risks are defined as the risks of disease induction other than cancer due to excessive exposure to heavy metals^23^. In this study, non-cancer health risk was calculated by using methods described by USEPA^9^ and Qu *et al*.^34^. This method is based on metal’s average daily intake (ADI) by the workers through exposure to a specific medium (indoor dust in this study). The following Eq. 2 is used to calculate the ADI, which probes the time-based association between average doses of certain heavy metal to its exposure level.

2 $$ADI=\frac{C\times IR\times EF\times ED\,}{BW\times AT}$$

where ADI express the average daily intake/dose of heavy metal (mg/kg/day) *via* dust exposure through different routes (inhalation or ingestion); C is the chemical concentration of metal in the medium *i.e*. dust (mg/kg); IR is the rate of ingestion (kg/day); EF represent the frequency of exposure (day/year); ED stands for duration of exposure (years) to a specific medium; BW is the body weight (kg) of the worker; AT is the average time period. Further, the risk posed to the exposed workers was quantified as Hazard Quotient (HQ) using ADI values from Eq. 2. Finally, the HQs of heavy metals were calculated using following equation Eq. 3.

3 $$HQ=\frac{ADI}{RfD}$$

where ADI express heavy metal’s average daily intake (mg/kg/day) calculated using equation (1) and RfD represent the oral reference dose of certain metal extracted from the database and listed in Table S9. The HQ values <1 are considered as harmless with no/minimal risks to the health of the exposed worker with that exposure level to certain heavy metal, while the values of HQ > 1 portray possibility of serious health risks, which can lead to the induction of disease. Moreover, Hazard index (HI) was calculated by adding up HQs for all the investigated metals, which indicated overall potential high-end health risk rather than a low-end risk. In this study, ADI and HQs were calculated for all the workers and biases regarding risk calculations were removed by applying Monte Carlo simulation method with 10,000 iterations through Crystal Ball software (Oracle Corporation, Vallejo, USA).

#### Cancer risk

Cancer risk was computed only for Cr (VI) and Cd using the following Eq. 4. as described by Xu *et al*.^35^ and US EPA (US EPA, 2007).

4 $$CR=CSF\times C(\max )$$

where CR is the risk of cancer incidences and CSF is the cancer slope factor of the specific carcinogenic metals. The values used for cancer slope factor are provided in the Table S9.

### Superoxide dismutase activity assay

SOD activity assay was used to quantify the oxidative stress mediated by heavy metals as described by Sun *et al*.^36^. Briefly, phosphate buffers with pH 7.8 and pH 7 were prepared. Polyvinylpyrrolidone (PVP), Ethylenediaminetetraacetic acid (EDTA), Methionine, Nitro blue tetrazolium chloride (NBT) and Riboflavin solutions were prepared following the standard procedure. An aliquot of 0.1 mL blood was centrifuged at 1500–2000 × g at 4 °C for 10 min. Reaction and reference mixtures were prepared following the same methods as mentioned above. Phosphate buffers and distilled water were used to prepared blanks. Afterward, the absorbance of the prepared samples and blanks was measured at 560 nm by using spectrophotometer Model DR5000 (HACH, USA). SOD activity was detected as reported by Beauchamp and Fridovich^37^.

## Results and Discussion

### Heavy metal concentrations and correlations in the biomatrices

**Figure 2:**
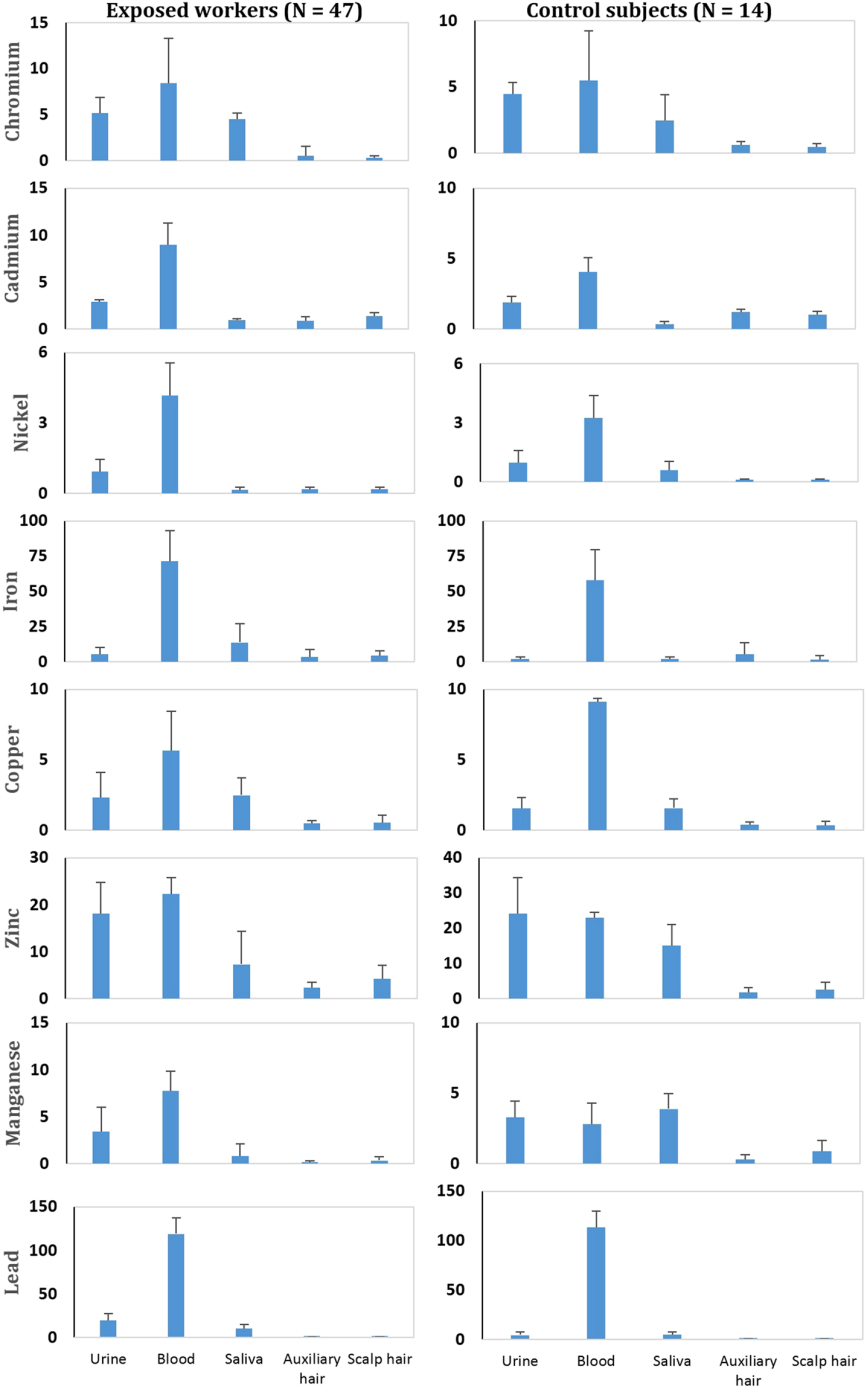
Heavy metal concentrations in the bio-matrices of exposed and control subjects in leather industries. Heavy metal concentrations in urine, blood, and saliva were measured in µg/L and in µg/g in hair samples.

The concentrations of heavy metals in the blood and urine samples of exposed worker and control group are presented in the Fig. 2. Cr levels in the urine and blood samples were reported higher in exposed workers as compared to that in the control group (5.16 µg/L *vs*. 4.47 µg/L in the urine and 8.39 µg/L *vs*. 5.48 µg/L in the blood). Similarly, the level of other heavy metals *e.g*. Fe (71.48 µg/L), Pb (119 µg/L), Mn (7.7 µg/L), Cd (9.0 µg/L), and Ni (4.18 µg/L) were also measured higher in the blood samples of the exposed group compared to that of the control group. Moreover, the heavy metals’ concentrations in saliva of the exposed workers *e.g*. Cr (0.93 µg/L), Cd (4.53 µg/L), Cu (2.49 µg/L), Fe (13.85 µg/L) and Pb (10.22 µg/L) were also reported higher to that of the control subjects. In the scalp hair samples, all of the metals (except Cr and Mn) showed higher concentrations than those reported in the auxiliary hairs within the exposed group. In the auxiliary hair samples, the concentrations of Cr, Cd, Fe, and Mn were lower in the workers compared to that of the controls. Spearman correlation coefficients of heavy metal concentrations in bio-matrices are presented in the Table S1. Cr exhibited significant positive correlation with Mn (r = 0.51) in the urine, with Pb (r = 0.58) and Cr (r = 0.48) in the blood, and with Cd (r = 0.34) in the hair samples. Cr in the saliva samples was also positively correlated with Ni (r = 0.28) and Cr (r = 0.62) in the urine, with Cr (r = 0.49), Ni (r = 0.28), and Fe (r = 0.34) in the blood. In the exposed workers, Pb content also revealed significant positive correlations, such as Pb in the blood with Mn in the urine (r = 0.27) and Cr in the blood (r = 0.28). The levels of Pb in the saliva samples of the workers were also positively correlated with Cd in the urine (r = 0.27) and blood samples (r = 0.32), and also with Zn (r = 0.33) and Mn (r = 0.34) in the blood samples. Moreover, PCA analysis also showed similar results as those determined by the correlation matrix. PCA didn’t explain the high variance of the metal content in the bio-matrices. For example, the heavy metal concentrations in the urine, blood, saliva and hair samples of the exposed *vs*. control groups as variables explained 54, 59, 56 and 58% variances in the cumulative metal exposure index. Factor loading variables of PCA are presented in the Table S4–S8.

### Industrial sources of heavy metals

**Figure 3:**
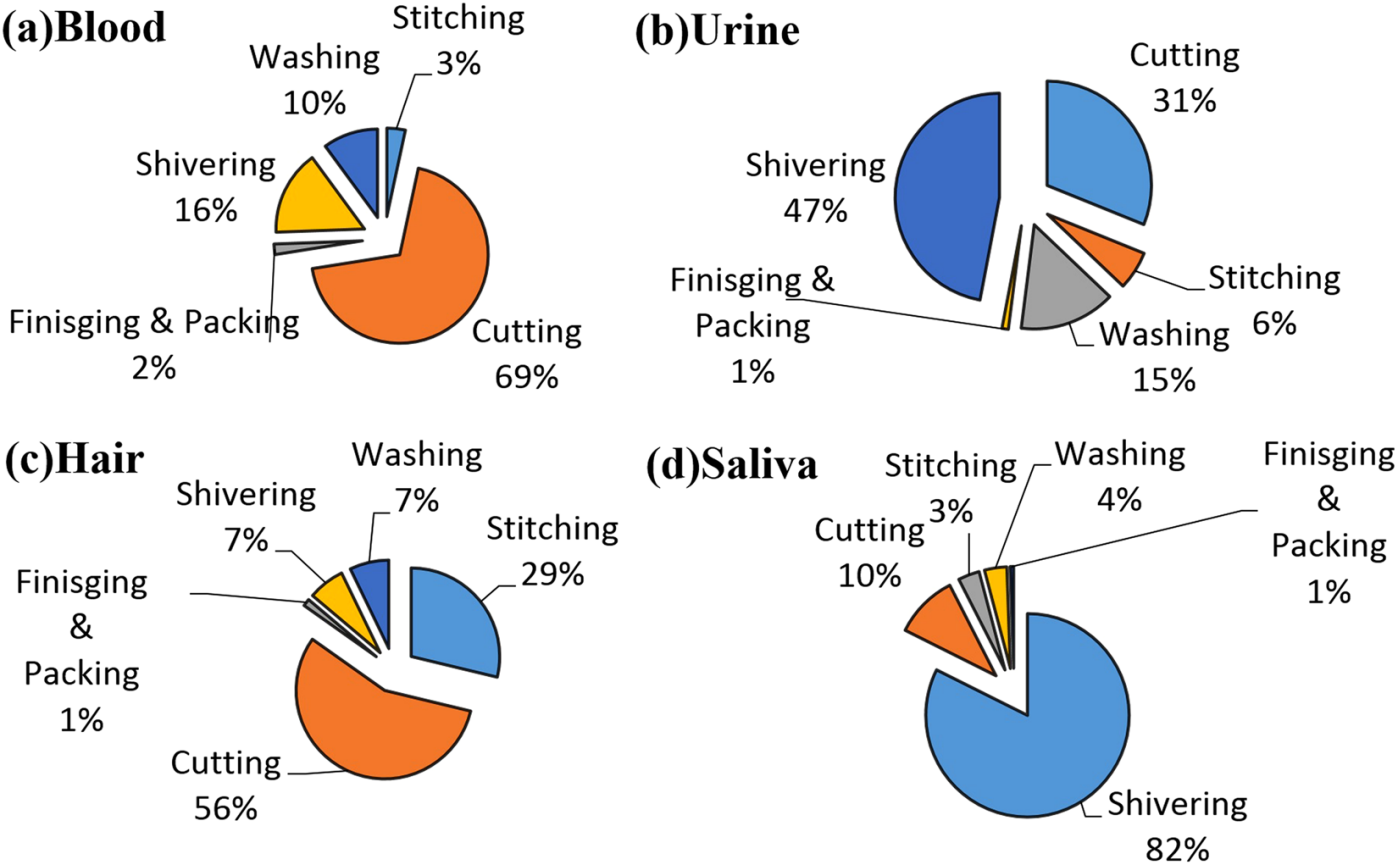
PCA-MLR based percentage contributing sources for the contamination of heavy metals in different biological matrices of occupationally exposed workers in the leather industries of Sialkot, Pakistan.

Workers were exposed to different levels of heavy metals in various sections of the leather industries *via* dust and fiber wastes. Therefore, to quantify the contribution of each of these sections in the heavy metal burden of the bio-matrices, we used a contemporary extended statistical technique known as PCA-MLR. The regression coefficients were computed among overall parameters (all the PCA axes were extracted from the purposed industrial sources, such as shivering/crusting, washing, cutting, stitching, and packing/finishing) and leave parameter (excluded PCA axis, such as shivering, washing, *etc*.) for the regression analysis. Regression analysis resulted in overall significantly higher coefficients (*R* ^2^ ≥ 0.87, 0.96) for blood and hair samples while lower for saliva and urine samples. The regression coefficients had the reliable competence between the predicted and measured values. Generally, regression values (*R* ^2^) higher than 0.75 are suitable for using this technique, which implied a good fit model among the measured and predicted heavy metal levels. The final percentage contribution of each source was calculated by using Eq. 1. As shown in the Fig. 3, pie charts were used to display the percentage contribution of five potential sources of heavy metal contamination of the bio-matrices in the exposed workers. The dust samples from shivering/crusting section showed the highest contribution of heavy metals in the saliva and urine samples (82% and 47%, respectively) while cutting section contributed 69% heavy metals in the blood samples and 56% in the hair samples. Stitching and washing section on average has the contribution of 3–29 and 10–15% in all bio-matrices, respectively, while packing and finishing sections of leather goods have a negligible contribution to heavy metal contamination of biological matrices.

### Heavy metals mediated health risk assessment

#### Non-cancer risk

**Figure 4:**
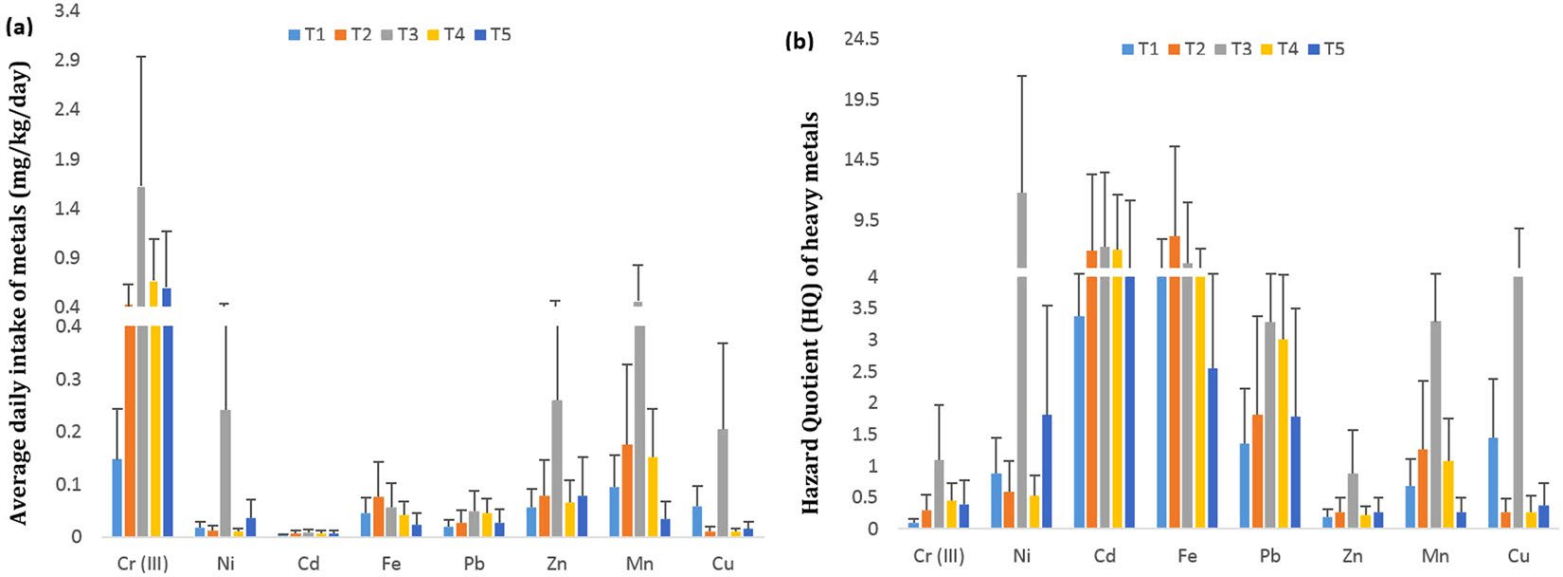
Health risk assessment associated with heavy metals intake through industrial dust exposure. (**a**) Average daily intake of metal (ADI), (**b**) hazard quotient (HQ) of heavy metals in the exposed workers from five different leather industries (T1, T2, T3, T4 and T5) of Sialkot, Pakistan. Note: HQ for Cr(VI) was reported highest among all the heavy metals in this study, due to large values of HQs for Cr(VI), they were not included in this chart to make this figure representable.

**Figure 5:**
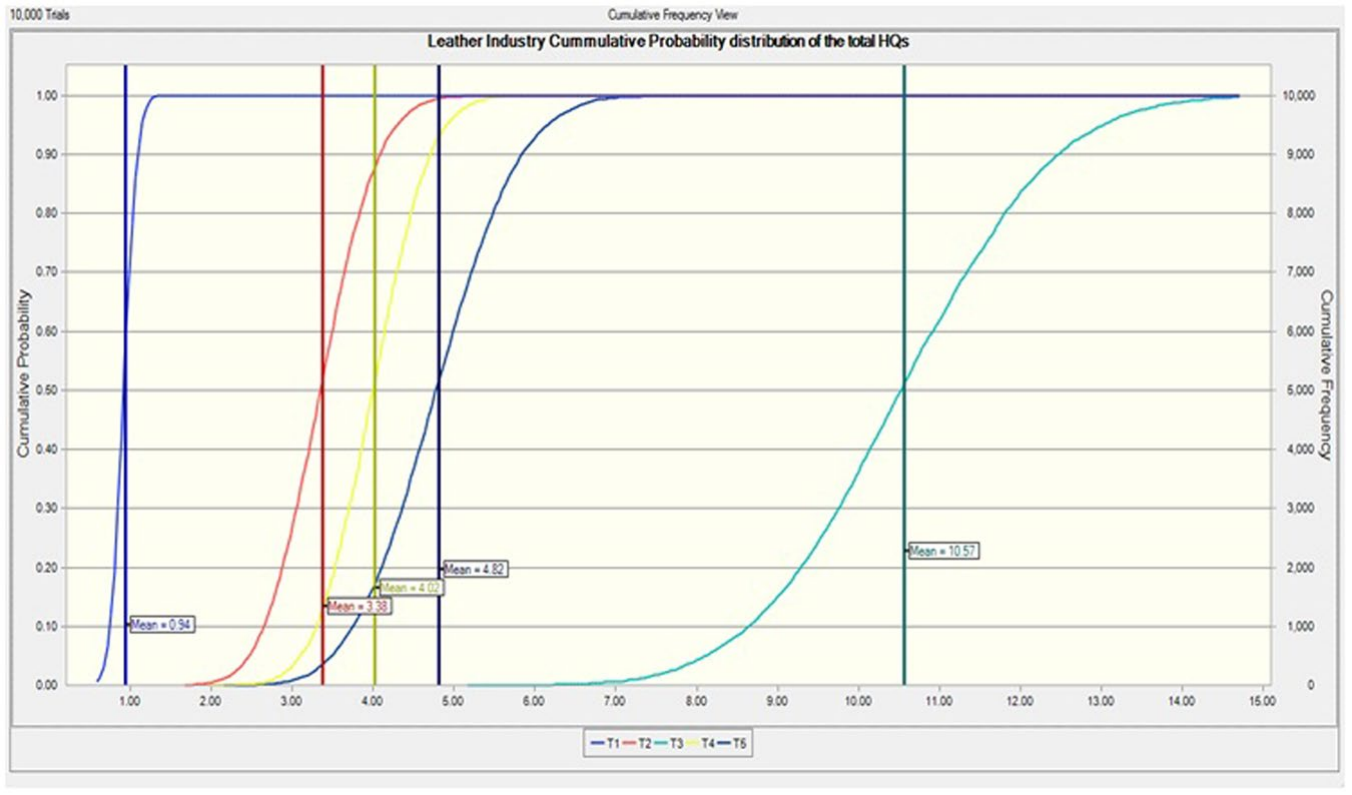
Cumulative Probability Distribution (CPD) of the total HQs in all five leather industries. The results for health risks to the exposed workers were validated using the Monte Carlo simulation based on crystal ball software (Oracle, USA) for 10,000 iterations.

To specify the daily intake frequencies of the indoor industrial dust and related health risks, we calculated the chemical daily intake (CDI), hazard quotient (HQ), and hazard index (HI) of investigated heavy metals through the intake of indoor industrial dust by the exposed workers. The comparative results of ADI and HQ of the investigated metals for the exposed workers in five leather industries are presented in the Fig. 4. Oral reference dose (RfDs) of heavy metals used for the calculation of HQ are provided in the Table S9. Figure 5 indicated the cumulative probability distribution curves plotted by Monte Carlo simulation (10,000 iterations) based on the total hazard quotient values of five leather industries.

The HQ values of Cr(III) for workers belong to all the investigated industries showed a narrow range and corresponding ADI values were also less than the respective RfD except in the leather industries T3 and T5, where ADIs of Cr(III) were 2–3 fold higher than the RfD. The mean highest HQ for Ni was measured in the workers of the leather industry T3 (12.08) with mean ADI of Ni at ﻿0.24 mg/kg-d. However, the ADIs of Ni in the workers of the remaining industries were lower than the RfD. In the same leather industry (T3), the highest mean HQs for Cd and Fe were calculated as 7.68 and 5.02 and the ADIs were 0.008 and 0.077 mg/kg-d, respectively. Compared to RfDs, the ADIs for both of the metals were almost 7-8 fold higher. Pb revealed the highest mean HQ of 3.28 with an estimated ADI of 0.049 mg/kg-d, which was about 300 folds higher than that of Pb’s RfD (0.00014 mg/kg-d). The mean HQs for Zn, Mn, and Cu were 0.87, 3.29, and 4.96, and their ADIs were 0.26, 0.46, and 0.20 mg/kg-d, which were 2, 4, and 5 fold higher than the corresponding RfDs (Table S9). The mean HQ value of Cr(VI) was about 134.50 with the ADI value of 0.67 mg/kg-d, which was about 120 fold higher than that of the RfD for Cr(VI).

#### Cancer risk of Cr and Cd

The cancer risks of Cd and Cr in the exposed workers were calculated using the specifically devised cancer slope factor (CSF) values. The CSF values used in the current study are present in the Table S9. Cr expressed elevated cancer risks for the exposed workers compared to that of Cd (0.300 vs. 0.041), albeit Cd showed elevated risk than that of Cr (6.87 vs. 0.11) for non-cancer risk.

### Heavy metals induce oxidative stress in the exposed workers

The SOD level in the exposed group was significantly higher (*p* < 0.01) than that in the control group (Table 1). Correlation analysis indicated significant positive association (*p* ≤ 0.01) among SOD levels and concentrations Cd, Cr and Zn in the blood with the following order: Cr > Zn > Cd for most of the exposed workers. However, no significant association revealed between SOD concentration, age, and exposure duration for the workers, albeit control subjects implied significant positive correlation for these independent variables (*p* < 0.01). Moreover, adjusting the marital status, working time span, and smoking, the linear regression coefficients showed that SOD levels in blood were significantly dependent on Cr (r = 0.74), Cd (r = 0.92), and Zn (r = 0.88).

**Table 1:** SOD concentration (mean ± SD), correlation and regression analysis among the blood SOD levels, heavy metals, age and working exposure in the exposed groups and control.

SOD level (units-g^−1^)	Exposed workers (134.59 ± 35.170)			Unexposed subjects (108.29 ± 6.10)		
	Heavy metal conc.(µgL^−1^)	Corr.	r^2^	Heavy metal conc.(µgL^−1^)	Corr.	r^2^
Cd	8.39 ± 4.87	−0.391	0.016	5.48 ± 3.75	1.000**	0.918**
Cr	9.00 ± 2.30	0.927**	0.745**	4.05 ± 1.03	0.200	0.625**
Ni	4.18 ± 8.37	0.551*	0.562*	3.25 ± 1.13	0.800**	0.480
Fe	71.48 ± 21.68	−0.527*	−0.312	57.99 ± 21.70	−1.000**	−0.932**
Cu	5.63 ± 2.79	−0.127	−0.188	9.10 ± 0.27	0.211	0.327
Zn	22.30 ± 3.46	0.945**	0.775**	22.97 ± 1.50	0.400	0.621**
Pb	119.04 ± 59.32	0.436	0.267	113.3 ± 16.83	−0.400	−0.123
Mn	7.77 ± 11.1	0.210	0.072	2.82 ± 1.46	−0.800**	−0.877**
Age (years)	34.47 ± 14.38	−0.498	−0.322	27.75 ± 9.18	1.000**	0.997**
Time span (years)	14.00 ± 8.73	−0.056	0.412	6.00 ± 3.65	0.800**	0.775**

Corr. = Correlation matrix, r = linear regression *p ≤ 0.05, **p ≤ 0.01.

### Statistical evidence for the transfer of metals from dust to human body

To elucidate the impact of contaminated indoor industrial environment on the exposed workers, correlation analysis was conducted between the heavy metal footprints in the dust *vs*. those reported in the saliva and urine samples (relatively directly exposed excretory fluids). The results revealed that Cr exhibited a significant positive association between saliva and dust samples (r = 0.40), implying the transportation of Cr from indoor industrial dust to the saliva of the exposed workers through oral cavity (Table S10). The elevated concentrations of Cr in the dust samples also strongly affected other heavy metals, *e.g*. Cr in the dust samples was positively correlated with Cu in the saliva samples (r = 0.61). The level of Pb in the saliva and dust samples showed significant positive correlation (r = 0.57). The Cr concentrations in the urine samples were negatively correlated with Ni in the dust samples (r = −0.40). However, Ni concentration was lower than other metals. Cd in the urine samples and Mn in the dust samples showed a positive relationship with Ni (r = 0.60). Pb in the urine samples was also significantly linked with Fe (r = 0.40) and Ni (r = 0.49) in the dust matrix (Table S11).

## Discussion

Occupational exposure to elevated levels of heavy metals, associated oxidative stress, and health risks in exposed workers have been reported previously in leather industries worldwide^38–42^. However, this is a robust study from Sialkot (the largest Asian exporter of leather products), which included simultaneous biomonitoring of eight heavy metals in five biomatrices, their industrial sources, associated oxidative stress, and health risks in exposed workers of the leather industries. This study discovered the elevated level of toxic heavy metals (Cr, Cd, Pb, and Ni) and compromised levels of essential metals (Zn, Mn, Fe, and Cu) in exposed workers, compared to that of the control group. The other three major findings reported in this study are: (1) Cr (VI) and Ni poses the alarming level of non-cancer health risks to the workers and carcinogenic risk of Cr was higher than that of Cd. Consistently, these two metals (Cr and Ni) induced highest levels of oxidative stress in the exposed workers; (2) shivering/crusting, cutting, and stitching of leather were identified as the primary sources of heavy metals responsible for contaminating the biological matrices of the exposed workers; (3) Cr, Ni, Pb, Cu, Zn, and Mn levels detected in biomatrices (saliva and urine) and industrial indoor dust revealed strong positively correlation, implying direct transportation of these metals from indoor industrial dust to the biological fluids of the exposed workers.

Blood and urine samples are known as the classical biomatrices because of their frequent use in biomonitoring studies and high acceptance among the scientific community^25, 39^. Among classical bio-matrices, an elevated level of heavy metal was reported in the blood samples, compared to the urine samples. The reason could be the reduction of heavy metals to less toxic metallic ions in the liver and excrete through urinary tract within 8 hours of ingestion^12^. While heavy metals retain their oxidation state intact in the blood regulatory system for longer period of time^43^. Generally, the elevated level of Cr in all the bio-matrices can be attributed to the excessive use of Cr sulphate salts [Cr(H_2_O)_5_(OH)SO_4_] in leather tanning^2, 44^. However, Cr levels measured in the urine and blood samples of the exposed workers in the current study were lower than those reported in the previous studies from China^42^ and India^38^ (Table 2). The concentrations of Cd and Ni in the blood samples were higher than those reported in the worker from Spain (5.61 µg/L and 4.66 µg/L, respectively)^25^. In this study, Mn has ~2 fold higher concentration in urine samples than that of workers from Heidelberg, Germany^45^. The general reason behind higher levels in the occupational setting of Pakistan and lower in developed countries could be the unhygienic working conditions and lack of protective measures in Pakistan^46^. Higher concentration of Pb in the blood samples of the exposed workers and controls may be attributed their exposure to Pb-smoke and low-grade diesel from the industries. Although Pakistan has banned Pb-fuel in 2002, previously deposited Pb in the environment may contribute to the current occupational exposures^30^. We also quantified the Pb content in the industrial effluents and sediments from nullahs in the city; the Pb concentrations were ranged 6.42–7.72 mg/L and 15.5–13.4 mg/kg, respectively (Fig. S1). Interestingly, the concentrations of Cu and Zn in blood samples of the control group were higher than those in the exposed workers. According to Fraga, the estimated average requirements (EARs) of Cu and Zn are 0.7 and 8.1 mg/day for a healthy human body, respectively. The adequate level of these metals is also essential for the natural defense against the xenobiotics in the human body^47^. Therefore, the lower concentration of essential elements in the exposed group may be an indication of oxidative stress that compromised the adequate levels of the trace elements.

**Table 2:** Concentration level of heavy metals in the biological matrices of workers in different occupational settings from published literature.

Location	Subjects	Industry Category	matrix	Cr	Cd	Ni	Cu	Mn	Zn	Pb	Analytical methods	References
Sialkot, Pakistan	47	Leather tanning and products manufacturing	Urine	5.16	2.90	0.93	2.31	3.39	18.12	7.58	FAAS	This study
			Blood	8.29	9.00	4.18	5.63	7.77	22.30	119		
			Saliva	4.53	0.93	0.15	2.49	0.84	7.33	10.2		
			Auxiliary hair	0.56	0.86	0.18	0.5	0.14	2.30	0.56		
			Scalp hair	0.31	1.38	0.19	0.56	0.35	4.29	0.55		
Zhejiang, China	90	Leather Tanning	Urine	10.60							GFAAS	42
			Blood	22.95								
Kanpur, India	100	Leather Tanning	Blood	147.75							AAS	38
Camerino, Italy		Leather Tanning	Urine	1.86							AAS	40
			Blood									
Lahore, Pakistan	160	Carpet weaving brick kiln	Urine	0.38	0.36	3.33	11.5	1.5	169	3.60	ICP-MS	71
				0.38	0.54	4.33	15.8	2.08	411	8.52		
Gdansk, Poland	42	Slide bearings	Blood				783		5840	274	ICP-MS	72
			Urine				17.8		334	25.5		
Germany	92	Lead-smelter	Blood							500	ICP-MS	73
			Urine							25		
			plasma							2.7		
Spain	144	Steel	Saliva		0.14	7.8		6.94	3.0		ETAAS	74
China	49	Welding	Saliva		0.36		28.5	4.45	191	24.9	ICP-MS	16
United Kingdom	25	battery workers	Saliva		7.87						AAS	75
Canada	19		Saliva		59.7						AAS	76
Sweden	5	Divers	Saliva				10–200				ETAAS	77
China	122		Saliva					22.3			AAS	78
Canada	5	battery workers	Saliva							129	AAS	79
Syria	281	printing plants	Scalp hair			2.74	8.94		184	2.51	X-ray fluorescence	80
Pakistan	62	Steel Mill	Hair		2.53			5.89	270	8.93	AAS	81
			Blood		5.16			63.9	8.54	263		
			Urine		3.95			2.5	1.27	105		
Berlin Germany	28	incinerator	Blood					15.1		41.3	ICP-MS	82
			Urine		0.45	14.8						
Heidelberg, Germany	100	dry cell	Blood					10.7			GF-AAS	45
			Urine					0.26				
			Hair					4.6				
Umbria, Italy	19	Cr plating	Urine^a^	7.31							GFAAS	83
			Blood	50.30								
Granada, Spain	178	Iron and steel	Urine^a^	0.95	0.25	1.67		0.93		22.28	ETAAS	25
			Blood	1.31	0.49	0.96		8.68		43.39		
			Saliva	3.11	0.14	7.79		6.94		3.03		
			Hair	3.14	0.07	0.12		2.35		24.33		
Netherlands	53	Welding	Urine^a^	0.30							ICP-MS	84
			Blood	10								
Bochum, Germany	241	Welding	Urine	1.0							GFAAS	85
			Blood	1.5								
Jinan, China	115	Chromate	Blood	12.45							ETAAS	86
Sialkot, Pakistan	102	Surgical industry	Urine	23	0.48	7.45	16.3	3.33	278	4.27	ICP-MS	41
				30								
Sialkot, Pakistan	75	Surgical industry	Urine	58.15	41.38	12.04	2.53	5.57	215	10.15	FAAS	58
			Blood	16.3	5.61	4.66	1.52	2.08	13.5	25.18		
			Saliva	1.25	5.28	4.12	0.1	7.7	16.16	10.57		
			Auxiliary hair	2.9	1.06	0.08	0.43	0.14	2.01	0.37		
			Scalp hair	2.36	1.4	0.04	0.49	0.06	1.52	0.39		

Units: urine (µg/L, ^a^µg/g creatinine), blood & saliva (µg/L), hair (µg/g).

Saliva and hair samples are known as non-invasive biomatrices because of their infrequent use as biomonitoring tools and less acceptance, compared to the classical bio-matrices^25, 39^. Recently, the use of non-invasive bio-matrices have been increased significantly because of their potential to store higher metal content (hairs), the longer half-life of metal (hairs), higher willingness to donate samples, easy collection, and transportation^25, 39^. The concentration of metals in the saliva represent the recent, while in the hairs represent the long-term heavy metal exposure. In the current study, Cd, Mn and Pb concentration in the salivary samples were higher compared to the previous study^25^. While compared to our current findings, worker’s from Spain showed lower Cd level (0.07 µg/g) in the auxiliary hairs^25^. Overall, the heavy metals levels detected in the scalp hairs were higher than that of the auxiliary hair in the current study, and this might be an indication of external contamination in the scalp hairs as described previously in our study^39^ and by Gil *et al*.^25^. Hairs have an ability to accumulate 10-fold higher metal content than other bio-matrices^25, 48^. Therefore, hair samples expressed the highest concentration in the current study (Table 2). Moreover, the elevated metal content in the hair samples indicated the long-term occupational exposure to heavy metals^25^.

Cr is the primary metal of concern in leather industries, because it proved as one of the best leather tanning agent worldwide^4^. Albeit, abundance of Cr in the indoor industrial environment can cause serious health implications to the exposed workers because of its carcinogenic nature, and its capability to enter the body through various exposure pathways^49^. Cr belongs to the group of toxic metals, which has a small half-life in the soft tissues (blood and saliva) and readily excrete from the body through urination or reduction to the metabolites^12^. This might be the reason behind significant positive correlations among Cr and other metals in the soft tissues (urine, blood, and saliva) (Table S1). These positive correlations indicated the inter-matrices transportation of heavy metals in the exposed workers^39^. Moreover, heavy metals also have potential to transport between the body skeleton and blood, which is called “endogenous contamination”^43, 50^. Importantly, the Cr reduction metabolites have physiological resemblance with phosphate and sulfates, allowing their easy entry into the cells *via* non-specific anionic pathway and ultimately lead to the cellular DNA damage or failure of DNA replication^51, 52^.

Previous reports mentioned high possibility of endogenous contaminations for Cd and Pb because of their long half-lives in the human body, (10–40 years) and (2 months to 20 years), respectively^43, 50^. Therefore, the elevated contamination levels and significant correlations express by these metals in the current study may be due to the excessive use of these metals in insecticides, batteries, glazed pottery, fuels, PVC paints, plating, and smelting processes^53, 54^. Pb contamination in the tap water was also reported in the study area with an elevated level of 0.46 ppm, which was 46 fold higher than that of WHO permissible limits (0.01 ppm)^29^. Contrary to the classical biomatrices, the hair metal content failed to reveal significant correlations, but this is one of the suggested limitations of using hairs as biomonitoring tool^55^. The correlation analysis of metal concentration among hair samples and those in the blood or urine samples are not commonly used in occupational and general exposure assessments^39^. Because hair samples represent long-term, while blood/urine/saliva samples express recent heavy metals exposures, thus making it unlikely any obvious correlation exist among them. Taken together, elemental concentration (especially Cr, Pb, and Ni) expressed significant association with other heavy metals in different bio-matrices, except hairs. The differences among toxicokinetic properties of the bio-matrices may also account for the lack of correlations between hair and other bio-matrices^56, 57^.

On the basis of job function, the leather industries consist main five sections: washing, shivering/crusting, cutting, stitching, and packing/finishing in the leather industries^2, 58^. In agreement with our previous study on child labor^39^, the shivering or crusting section was the principal contributor of heavy metals in urine and saliva followed by cutting section in blood and hair samples of the exposed workers in the current study. The shivering/crusting sections of leather industry have different sub-sections, such as buffing, dyeing, sammying (drying), shaving, stuffing, softening, and washing of leather. Therefore, the shivering/crusting mostly produces gains/particulate fraction of leather dust. That’s why it was highly contaminated section of the leather industries, while cutting, stitching and packing sections were less polluted because they produce fiber waste.

The indoor dust from leather industries poses serious health risks to the exposed workers due to the excessive contamination of the heavy metals^39^. The fine fraction of the dust can transfer to the human body *via* different exposure pathways, such as ingestion, inhalation, and dermal contact^23, 24, 59^. According to the health risk classification of the USEPA, when the HI value of heavy metal exposure is >1, the associated risk becomes higher. Consequently, in the longer run, the probability of disease induction also becomes higher in the exposed population^60^. Unfortunately, the mean HI values were >1 for all the investigated workers from different leather industries with the order of T3 > T2 > T4 > T5 > T1. Leather industry T3 was located relatively closer to the urban area, therefore, workers maybe intake the additional burden of heavy metals from non-point sources. Monte Carlo simulation model also confirmed these results with a higher probability of health risks induction in the workers belong to the leather industry T3 as compared to other industries. The elevated footprints of heavy metals in the indoor industrial dust can cause serious health risks to the exposed workers in the following order: Cr(VI) > Ni > Fe > Cd > Cu > Pb ≈ Mn > Cr(III) > Zn. Since Cr (VI) is mutagenic and carcinogenic in nature, the RfD values of Cr (VI) were low. Therefore, higher health risks were revealed to the exposed workers. These findings were consistent with the previous reports, highlighting the risks of indoor dust exposure in an urban area of Istanbul, Turkey^23^ and street dust from metals smelting district Huludao, China^61^. In this study, the HQ values of Pb were also higher than the threshold limits suggested by Agency for Toxic Substances Disease Registry (ATSDR)^62^. Cd also showed high HQ values for all the investigated workers. The previous report showed that Cd has a high bioaccumulation potential, specifically in the kidneys^63^. Flora and Pachauri reported that chronic exposure of Cd can cause prostate, lung, kidney and pancreatic cancer^63^, and such cases were reported previously in an industrial area of north-east Belgium^64^. The acceptable or tolerable threshold limit values for cancer risk is ranged from 1 × 10^−6^ to 1 × 10^−4^ (US EPA, 2001). In the current study, the Cr and Cd expressed the corresponding values for cancer risks of 3.4 × 10^−1^ and 3.9 × 10^−2^ in the workers, respectively. Unfortunately, these computed cancer risks were much higher than the acceptable limit values devised by USEPA.

Oxidative stress is induced by heavy metals through the unbalanced generation of ROS^14, 65^. ROS naturally fight against the xenobiotics in the body (heavy metals *etc*.) and are involved in the cell signaling pathways^20^. However, the excessive ROS can lead to inflammation, premature aging, atherosclerosis, hypertension, diabetes, and even cancer^66^. SOD, an antioxidant enzyme and an excellent biomarker for cellular oxidative stress, is the first and most critical line of the natural immune system against ROS. Besides assisting the body in the immune system, previous evidence showed that SOD enzyme was involved in various diseases, such as Down’s syndrome, Lou Gehrig’s disease and premature aging^66, 67^. For the exposed group, SOD activity was reported higher in the exposed group, albeit it didn’t reveal a significant association with exposure duration and age (*p* > 0.05) of the workers. Khan *et al*. and Hassan *et al*. also reported similar finding for the workers exposed at leather tanneries and gasoline stations, respectively^22, 68^. For the control group in this study, a significant positive correlation was found between SOD activity, the age of the worker, and working duration (*p* < 0.05). Similar findings were reported previously by Nielsen *et al*.^69^. Zn concentration in the blood samples showed a positive correlation and regression coefficient with levels of SOD activity. Interestingly, Zn also acts as an antioxidant to protect the cells from oxidative stress through activating the metallothionein enzyme (ROS inhibitor) and inactivating the NADPH enzyme activity (ROS generator)^70^. Thus, Zn may synergistically act with SOD to reduce the oxidative stress. Hence, our study revealed strong evidence related to the transportation of heavy metals from the dust to the worker’s body *via* various routes (Table S10 and S11), which was further confirmed by the elevated health risks and oxidative stress caused by heavy metals to the exposed workers at the leather industries in Sialkot, Pakistan.

## Conclusion

This study concluded the increasing heavy metal pollution especially in the blood, urine, and hair samples of the exposed workers and indoor dust samples. The heavy metals Cr, Ni, Cd, and Pb were identified as the main culprits posing the highest health risks and inducing the oxidative stress in the workers. Shivering/crusting, cutting, and stitching of leather were highlighted as the highest heavy metals contributing sections in the bio-matrices of the exposed workers through ingestion of the contaminated dust. Further, this study concluded that the unsafe and unhygienic indoor environment contaminated with industrial dust make workers susceptible to high metal exposure.

## Electronic supplementary material

Supplementary information
