# Supplementary material for: Potential health risk of heavy metals in the leather manufacturing industries in Sialkot, Pakistan
Source: Sci Rep. 2017 Aug 18;7:8848. doi: 10.1038/s41598-017-09075-7 (PMC5562736; doi:10.1038/s41598-017-09075-7)
Supplement: Supplementary file 1 — Supplementary information [file 41598_2017_9075_MOESM1_ESM.doc]

**Supplementary Information**

**Potential health risk of heavy metals in the leather manufacturing industries in Sialkot, Pakistan**

Muhammad Junaid ^a,b,c^, Muhammad Zaffar Hashmi^d^, Yu-Mei Tang ^a,c^, Riffat Naseem Malik^b^*, De-Sheng Pei^a^*

^a^Chongqing Institute of Green and Intelligent Technology, Chinese Academy of Sciences, Chongqing 400714, China.

^b^Environmental Biology and Ecotoxicology Laboratory, Department of Environmental Sciences, Faculty of Biological Sciences, Quaid-i-Azam University, Islamabad 45320, Pakistan

^c^University of Chinese Academy of Sciences, Beijing 100049, China

^d^Department of Meteorology, COMSATS University, Islamabad 45320, Pakistan

^*^Corresponding authors.

Riffat Naseem Malik

E-mails: r_n_malik2000@yahoo.co.uk (R.N.M)

Muhammad Zaffar Hashmi

E-mail: hashmi_qau@yahoo.com

De-Sheng Pei

E-mails: [peids@cigit.ac.cn](mailto:peids@cigit.ac.cn) (D.S.P) and [deshengpei@gmail.com](mailto:deshengpei@gmail.com) (D.S.P)

**Contents**

**Text S1 Samples collection and processing**

**S1.1** Leather industries and sampled population

**S1.2** Collection of the blood and serum samples

**S1.3** Collection of urine samples

**S1.4** Collection of axillary hair samples

**S1.5** Collection of saliva samples

**S1.6** Collection, storage and pre-treatment of dust, effluents, and sediment samples

**S1.7** Samples digestion and preparation methodology

**S1.8** Digestion of effluent, dust and sediment samples

**S1.9** Preparation of serum, saliva and urine samples

**Table S1** Digestion conditions for different biological and environmental matrices

**TableS2** FAAS instrumental conditions and QA/QC parameters for the measurement of heavy metals

**Table S3** PCA variable loadings for urine metals in the workers of leather industry

**Table S4** PCA variable loadings for blood metals in the workers of leather industry

**Table S5** PCA variable loadings for serum metals in the workers of leather industry

**Table S6** PCA variable loadings for saliva metals in the workers of leather industry

**Table S7** PCA variable loadings for hair metals in the workers of leather industry

**Table S8** Oral reference doses and cancer slope factors of different heavy metals

**Figure S1.** Mean heavy metal concentration in the dust (a), sediments (b) and effluent (c) in five different leather industries.

**Text S1 Samples collection and processing**

**S1.1 Leather industries and sampled population**

Five random potential industrial units were selected for sampling of indoor dust and bio-matrices (blood, urine, saliva, auxiliary and scalp hair). A total of 47 healthy workers and 14 unexposed individuals of different age groups were selected for biological samples. The methods for sampling are described somewhere [^1^](#_ENREF_1).

**S1.2 Collection of blood and serum samples**

For whole blood, 3 mL venous blood sample was collected in EDTA-K (purple top 5 mL) using BD syringes. Blood samples were stored at -20°C until assayed after proper labeling of each sample.

**S1.3 Collection of urine samples**

The post-shift urine was collected after 5 consecutive working days from the exposed workers. For each sample, 30 mL urine was collected in the labeled metal free containers (Wang et al., 2012).

**S1.4 Collection of auxiliary hair samples**

For auxiliary hairs, the armpits of each worker were cleaned using neutral soap and double-distilled water, approximate 0.1 g hairs were cut from the auxiliary region by using ceramic scissor according to the description by Afridi *et al*. [^2^](#_ENREF_2). The hair samples were sealed in the pre-cleaned containers of polyethylene containers for the safe storage and transport. To remove the external contamination, the auxiliary hair samples was first washed through ultrasonic cleaning method using a detergent solution (Triton X-100, Merck, Germany). Afterward, Milli-Q water was used for rinsing to remove the detergent and then samples again subjected to ultrasonic cleaning in an ethanol solution (Merck, Germany) and finally washed with Milli-Q water.

**S1.5 Collection of saliva samples**

For saliva samples, the workers were requested to rinse their oral cavity for 1 min using 10 mL of sterilized distilled water to remove any prior impurities. Then, ~ 6 mL of unstimulated saliva samples were collected into polypropylene tubes immediately for 10 min with the mouth closed. Salivary samples were collected before breakfast in the morning. The first 1 mL of saliva samples were discarded and the remaining were immediately subjected to centrifugation at 900×g for 5 min to precipitate the cellular debris and finally stored at −20 °C for further analysis [^1^](#_ENREF_1).

**S1.6 Collection, storage, and pre-treatment of dust, effluents, and sediment samples**

For indoor industrial pollution monitoring, 25 indoor dust samples (5 samples per industry) were collected. Dust was manually collected by using a cleaning brush and dustpan from different sections of industry (separate brushes and pans were used for each industry) and stored in the pre-cleaned polyethylene bags for safe transfer to the laboratory.

Prior to effluent collection, sampling bottles were washed with metal-free soap, rinsed many times with distilled water, soaked in 10% HNO3 for 24 h and finally washed with deionized water. Triplicate effluent samples from each industrial unit were collected from point sources in labeled 1-L strong plastic bottles after 6 h of peak industrial activities. Carefully, 5mL of concentrated HNO_3_ was added to effluent samples immediately after collection to minimize metal adsorption to the walls of the plastic bottles. Composite samples were stored in an insulated cooler containing ice and delivered to the cold storage facility at 4◦C to prevent any change in the effluent’s chemical nature until further analysis.

Surface sediment samples were collected from all the five leather industries. The upper 0–10 cm depths of sediments were randomly collected from beneath a shallow aqueous layer using a trowel or scoop. Samples were stored in pre-cleaned plastic bags after labeling and transferred to the laboratory for analysis.

**S1.7 Samples digestion and preparation methodology**

**S1.7.1 Digestion of blood and hair samples**

Digestion of blood and hair samples was performed according to the protocol described by Samanta *et al*. [^3^](#_ENREF_3). Mars 5 (CEM Corporation, version 194A02, USA), a microwave based digestion system with a rotor containing sixteen vessels was utilized. The digestion conditions for hair and blood samples are given in the **Table S1**. For digestion of hair samples, 0.5 g dry hair was put into a pre-cleaned and acidified Teflon digestion vessel. Then, 3 mL of 65% HNO_3_ and 1 mL of H_2_O_2_ were carefully added to vessels and put in the rotor and subjected to the digestion. The digest was kept for cooling and then to filtered using Whatman filter paper no. 42 into a 50 mL volumetric flask. Then, the filtrate was stored in the prewashed acidified polyethylene bottles for subsequent elemental quantification. Prepared samples and blanks were stored at 4 ºC and analyzed within 2 weeks. For digestion of the blood samples, 0.5 mL volume of whole blood sample took in a 25 mL PTFE tubes, and then added 2 mL mixture of concentrated HNO_3_ and H_2_O_2_ (2:1 v/v) to each tube and kept overnight for homogenization and subjected to the digestion process [^4^](#_ENREF_4). The digests were kept for cooling in the vessels and then filtered using Whatman filter paper no. 42 and raise the final volume up to 20 mL using double distilled water in a volumetric flask. The prepared blanks and samples were stored in pre-washed and acidified bottle at 4ºC for further heavy metal analysis.

**S1.7.2 Digestion of the dust, effluents and sediment samples**

The same digester Mars 5 (CEM Corporation, version 194A02, Matthews, USA) was utilized for the digestion of dust, effluents, and sediments samples. 5 ml of effluents and 2g of each dust and sediments were taken in clean acidified Teflon vessels and 8 ml Aqua Regia was added in both effluent and sediment samples and 4 ml in the dust, according to methods describes by US EPA (3005A US EPA) and Divrikli *et al*. [^5^](#_ENREF_5). Finally, the samples were subjected to digestion as per conditions given in the **Table S1.**

**S1.8 Preparation of saliva and urine samples**

Saliva samples were processed as per protocol described by Chirila & Draghici [^4^](#_ENREF_4). About 5 mL of saliva sample was taken in a flask and added 10 mL of 2 % HNO_3_. Then, the saliva solution was poured carefully into the pre-washed and dry Teflon vessels and finally vessels put in the rotor and subjected to Mars 5 for digestion. After the digestion, filtration was performed and raised the final volume of the filtrate up to 20 mL. The procedural blanks were also prepared to probe the contamination using the same methodology. Samples and blanks were kept at 4 ºC prior to analysis. The frozen urine samples were first thawed and then prepared following the methods described somewhere[^4^](#_ENREF_4). The urine samples were filtrated through the Whatman filter paper no. 42. A 5 mL volume of filtered urine sample was taken in a 30 mL volumetric flask, and then diluted 5 times with HNO_3_ (2 % solution in distilled water). Procedural blanks were also run alongside to evaluate the degree of contaminations during sample preparation. Finally, samples and blanks were stored at 4 ºC prior to the heavy metals quantification.

**Table S1** Spearman correlation analysis of the heavy metal concentrations in the different biological samples

| ** Correlation is significant at the 0.01 level (2-tailed). |
| --- |
| * Correlation is significant at the 0.05 level (2-tailed). |

U-Urine, B-Blood, H-Auxiliary and Scalp hair, S-Saliva

**Table S2** Digestion conditions for different biological and environmental matrices

| Matrix | Power  (Watts) | Temperature  (ºC) | Pressure  (Psi) | Start-up time (Min) | Holding time (Min) | Reference |
| --- | --- | --- | --- | --- | --- | --- |
| Blood | 300 | 120 | 800 | 15 | 15 | [^4^](#_ENREF_4) |
| Hair | 300 | 180 | 800 | 10 | 10 | [^4^](#_ENREF_4) |
| Dust | 600 | 160-180 | 350 | 20 | 20 | 3052-US EPA |
| Effluent | 600 | 180 | 450 | 15 | 15 | [^6^](#_ENREF_6) |
| Sediment | 600 | 180 | 450 | 20 | 20 | [^7^](#_ENREF_7) |

**Table S3** FAAS instrumental conditions and QA/QC parameters for the measurement of heavy metals.

| **Metals** | **Wavelength (nm)** | **Slit width (nm)** | **Flame type** | **LOQs**  **(µg/L)** | **LODs**  **(µg/L)** | **Accuracy**  **(%)** | **Precision**  **(%)** | **Recovery**  **(%)** |
| --- | --- | --- | --- | --- | --- | --- | --- | --- |
| **Cd** | 228 | 0.5 | Air/Acetylene | 0.143 | 0.0533 | 0.99 | 3.52 | 94.22 |
| **Cr** | 375 | 0.5 | Air/Acetylene | 0. 031 | 0.0232 | 1.67 | 3.51 | 100.76 |
| **Ni** | 232 | 0.2 | Air/Acetylene | 0.1322 | 0.05423 | 5.48 | 4.16 | 105.47 |
| **Cu** | 324 | 0.5 | Air/Acetylene | 0.0971 | 0.0315 | 4.27 | 3.80 | 97.06 |
| **Fe** | 248 | 0.2 | Air/Acetylene | 0.1546 | 0.0536 | 1.87 | 3.04 | 96.40 |
| **Pb** | 217 | 1 | Air/Acetylene | 0.1384 | 0.03246 | 0.99 | 2.77 | 100.22 |
| **Zn** | 213 | 1 | Air/Acetylene | 0.1546 | 0.0646 | 2.04 | 2.69 | 84.42 |
| **Mn** | 254 | 1 | Air/Acetylene | 0.0876 | 0.0378 | 2.48 | 2.86 | 100.34 |

LOQs and LODs were calculated using mean heavy metal concentration detected in the blanks plus 3 and 10 standard deviations, respectively. Accuracy, precision, and recovery values are based on average of three replicates. Accuracy values are presented in percentages as a deviation from the certified values.

**Table S4.** PCA variable loadings for urine metals in the workers of leather industry

| Heavy metals | Axis 1 | Axis 2 | Axis 3 |
| --- | --- | --- | --- |
| Cd | 0.494 | -0.05 | 0.366 |
| Cr | -0.565 | -0.338 | 0.053 |
| Ni | -0.378 | 0.133 | -0.054 |
| Fe | -0.039 | 0.746 | 0.221 |
| Cu | 0.119 | -0.102 | -0.701 |
| Zn | -0.023 | -0.432 | 0.547 |
| Pb | -0.193 | 0.332 | 0.117 |
| Mn | -0.491 | 0.042 | 0.09 |

**Table S5.** PCA variable loadings for blood metals in the workers of leather industry

| Heavy metals | Axis 1 | Axis 2 | Axis 3 |
| --- | --- | --- | --- |
| Cd | -0.25 | -0.112 | 0.569 |
| Cr | 0.373 | 0.531 | -0.026 |
| Ni | -0.12 | 0.18 | 0.619 |
| Fe | 0.431 | -0.449 | -0.022 |
| Cu | -0.449 | -0.329 | -0.155 |
| Zn | 0.379 | -0.588 | 0.217 |
| Pb | 0.41 | 0.089 | -0.119 |
| Mn | 0.292 | 0.096 | 0.455 |

**Table S6.** PCA variable loadings for serum metals in the workers of leather industry

| Heavy metals | Axis 1 | Axis 2 | Axis 3 |
| --- | --- | --- | --- |
| Cd | 0.314 | -0.559 | -0.207 |
| Cr | 0.135 | -0.39 | 0.594 |
| Ni | -0.224 | 0.472 | -0.305 |
| Fe | 0.512 | 0.239 | 0.085 |
| Cu | -0.209 | 0.264 | 0.6 |
| Zn | 0.439 | 0.319 | 0.234 |
| Pb | -0.35 | 0.019 | 0.3 |
| Mn | 0.461 | 0.289 | 0.002 |

**Table S7.** PCA variable loadings for saliva metals in the workers of leather industry

| Heavy metal | Axis 1 | Axis 2 | Axis 3 |
| --- | --- | --- | --- |
| Cd | -0.174 | -0.029 | -0.648 |
| Cr | 0.514 | 0.242 | 0.12 |
| Ni | -0.528 | -0.187 | 0.154 |
| Fe | -0.361 | 0.381 | 0.065 |
| Cu | -0.328 | -0.545 | -0.03 |
| Zn | -0.254 | 0.367 | 0.375 |
| Pb | -0.059 | -0.192 | 0.612 |
| Mn | -0.348 | 0.54 | -0.148 |

**Table S8.** PCA variable loadings for hair metals in the workers of leather industry

| Heavy metals | Axis 1 | Axis 2 | Axis 3 |
| --- | --- | --- | --- |
| Cd | 0.367 | 0.212 | 0.519 |
| Cr | -0.277 | 0.146 | 0.485 |
| Ni | -0.511 | 0.271 | -0.161 |
| Fe | 0.203 | 0.262 | -0.088 |
| Cu | 0.434 | 0.28 | 0.068 |
| Zn | -0.515 | 0.384 | 0.119 |
| Pb | -0.125 | -0.701 | -0.004 |
| Mn | 0.134 | 0.274 | -0.666 |

**Table S9** Oral reference doses (RfD) and cancer slope factors (CSF) of different heavy metals used in this study to calculate the carcinogenic and non-carcinogenic risks.

| Metal | RfD (mg/kg-d) | Source | CSF | Source |
| --- | --- | --- | --- | --- |
| Cd | 1.0E0-3 | IRIS, USEPA | 6.1E+0 | USEPA |
| Cr (III) | 1.5E +0 | IRIS, USEPA | 5.0E-1 | USEPA |
| Cr (VI) | 5.0E0-3 | IRIS, USEPA |  |  |
| Cu | 4.0E-2 | HEAST |  |  |
| Ni | 2.0E-2 | ATSDR |  |  |
| Pb | 1.4E-4 | IRIS, USEPA |  |  |
| Zn | 3.0E-1 | IRIS, USEPA |  |  |
| Mn | 1.4E-1 | IRIS, USEPA |  |  |

**Table S10** Correlations of heavy metal concentrations in the salivary samples from the exposed workers and indoor industrial dust


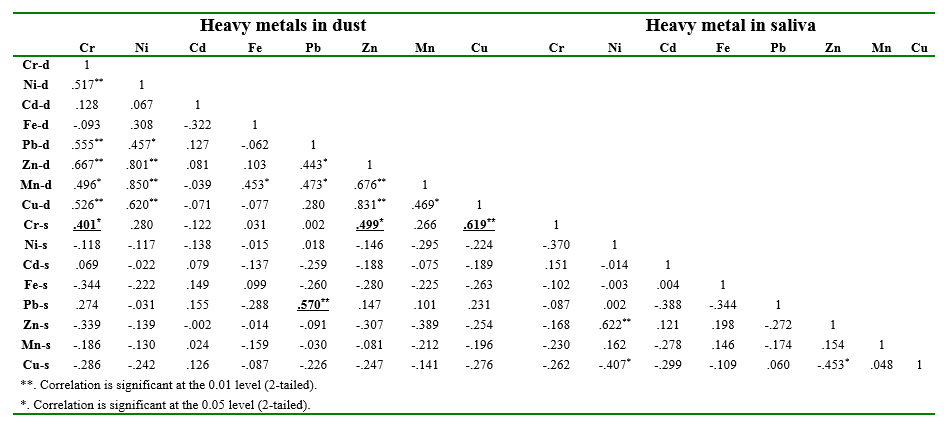


**Table S11** Correlations of heavy metal concentrations in the urinary samples from the exposed workers and indoor industrial dust


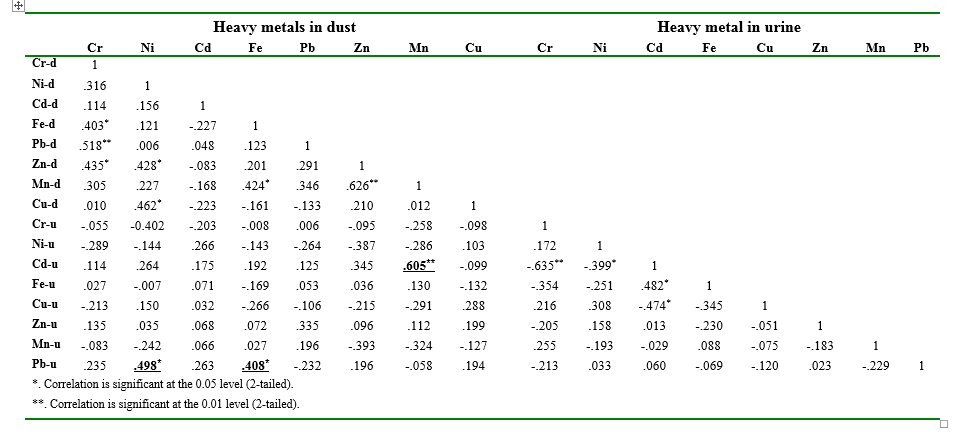


**
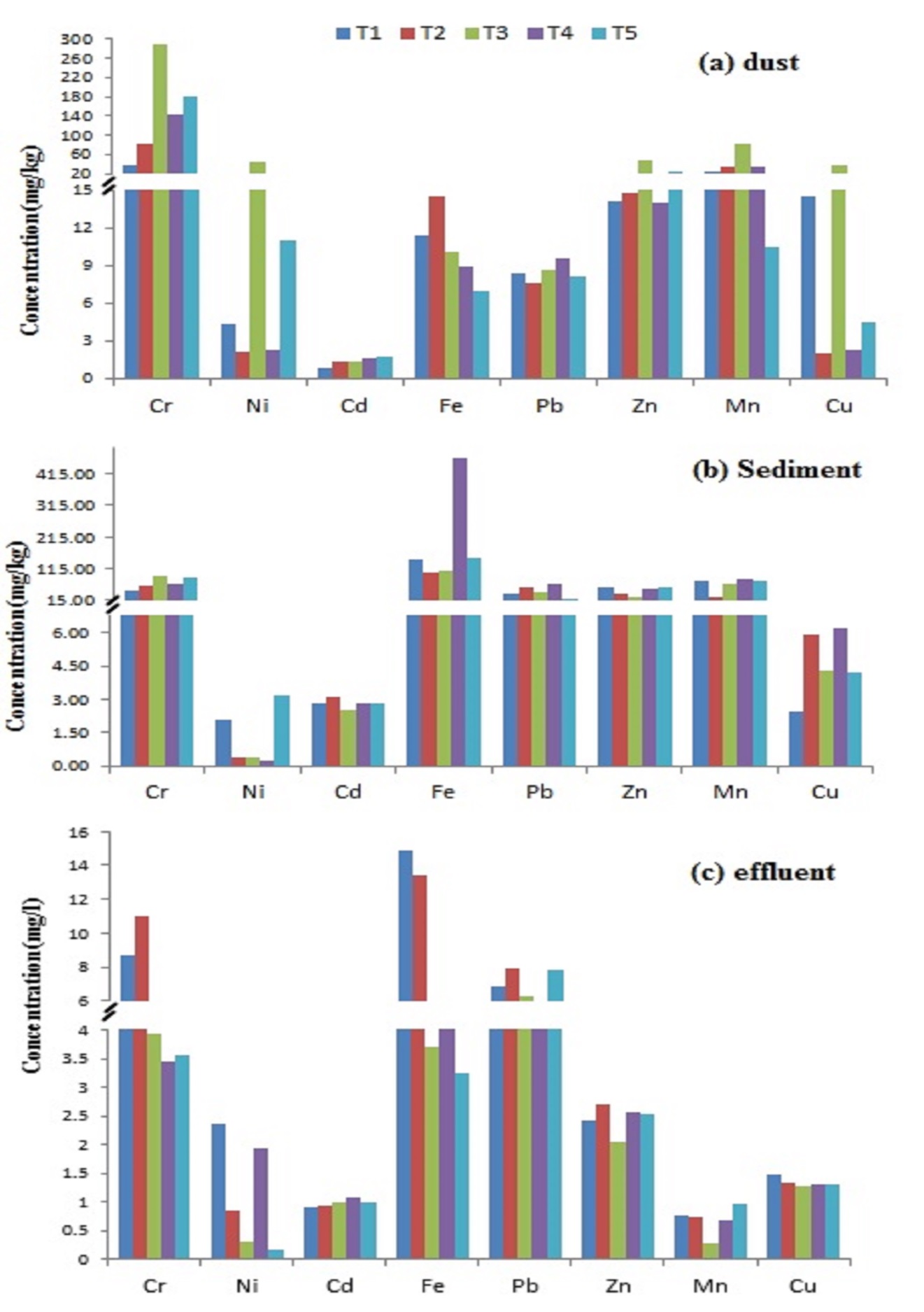
**

**Figure S1.** Mean heavy metal concentration in the dust (a), sediments (b) and effluent (c) in five different leather industries.

**References**

1 Gil, F. *et al.* Biomonitorization of cadmium, chromium, manganese, nickel and lead in whole blood, urine, axillary hair and saliva in an occupationally exposed population. *Science of the total environment* **409**, 1172-1180 (2011).

2 Afridi, H. I. *et al.* Levels of arsenic, cadmium, lead, manganese and zinc in biological samples of paralysed steel mill workers with related to controls. *Biological trace element research* **144**, 164-182 (2011).

3 Samanta, G., Sharma, R., Roychowdhury, T. & Chakraborti, D. Arsenic and other elements in hair, nails, and skin-scales of arsenic victims in West Bengal, India. *Science of the Total Environment* **326**, 33-47 (2004).

4 Chirila, E. & Draghici, C. in *Environmental Heavy Metal Pollution and Effects on Child Mental Development* 129-143 (Springer, 2011).

5 Divrikli, U., Soylak, M., Elci, L. & Dogan, M. Trace heavy metal levels in street dust samples from Yozgat City Center, Turkey. *Journal of trace and microprobe techniques* **21**, 351-361 (2003).

6 Nickel, B. *et al.* ACID DIGESTION OF WATERS FOR TOTAL RECOVERABLE OR DISSOLVED METALS FOR ANALYSIS BY FLAA OR ICP SPECTROSCOPY. (1992).

7 Paar, A. *Microwave Sample Preparation System’ –Instruction Handbook*. 128 (Anton Paar GmbH 1998).
